# Inversion Theory Leveling as a New Methodological Approach to Antioxidant Thermodynamics: A Case Study on Phenol

**DOI:** 10.3390/antiox12020282

**Published:** 2023-01-27

**Authors:** Nikola Stamenković, Janez Cerkovnik, Nataša Poklar Ulrih

**Affiliations:** 1 Department of Food Science and Technology, Biotechnical Faculty, University of Ljubljana, 1000 Ljubljana, Slovenia; 2 Department of Chemistry and Biochemistry, Faculty of Chemistry and Chemical Technology, University of Ljubljana, 1000 Ljubljana, Slovenia

**Keywords:** DFT, inversion theory leveling, antioxidants, thermodynamics, antioxidant acitivity, phenol

## Abstract

Antioxidants are various types of compounds that represent a link between biology and chemistry. With the development of theoretical and computational methods, antioxidants are now being studied theoretically. Here, a novel method is presented that aims to reduce the estimated wall times for DFT calculations that result in the same or higher degree of accuracy in the second derivatives over energy than is the case with the regular computational route (i.e., optimizing the reaction system at a lower model and then recalculating the energies at a higher level of theory) by applying the inversion of theory level to the universal chemical scavenger model, i.e., phenol. The resulting accuracy and wall time obtained with such a methodological setup strongly suggest that this methodology could be generally applied to antioxidant thermodynamics for some costly DFT methods with relative absolute deviation.

## 1. Introduction

Antioxidants as chemical species that serve to prevent oxidation processes (in the broadest sense) in living biological systems have evolved considerably in recent years, both in terms of experimental [1,2,3,4,5,6,7,8] and theoretical contributions [9,10,11,12,13]. The theory of antioxidants, which was introduced to explain all the related effects that antioxidants might have in biological systems, is usually considered to be the result of experimental improvements over the years. Nevertheless, viewpoints considered important in the study of antioxidants are related to the development of their chemistry. Therefore, current investigations are directed toward the study of their topography, synthesis, and microchemical (quantum) properties. All of the above chemical aspects are closely interrelated, and the development of only one of them would drive the other two in the same direction.

Experimental methods for characterizing antioxidants are diverse in terms of their structure or reactivity, and it should be additionally noted that the reactivity of antioxidants is usually defined as the overlap between their acidity, scavenging capacity, and nucleophilicity. Two-dimensional nuclear magnetic resonance (2D NMR) [14,15,16,17] (attenuated total internal reflection), Fourier-transformed infrared spectroscopy (ATR-]FTIR) [18,19,20,21], and colorimetric-based spectroscopy (the most appropriate method is UV/Vis) [22,23,24,25] are sufficient methods to determine the structure and content of antioxidants. However, it is advantageous to add that these methods are usually well combined with chromatographic methods and mass spectrometry (MS) [22,26] to properly determine antioxidant activity and perform mechanistic analyses of how antioxidants act at various biological sites. In addition, despite their popularity in characterizing compounds in the broader chemical community, X-ray techniques (XRD and XRF) [24,27,28] have occupied a less important place in the characterization of antioxidants, most likely because they are applicable only in strictly structure-based areas. In addition to structural characterization, the reactivity of antioxidants is a ubiquitous question for which an answer is constantly sought. This phenomenon, as mentioned earlier, involves the consideration of two general parameters that are actively involved in the outcome of the reactivity pattern: absolute acidity and radical scavenging capacity. Absolute acidity is defined as the ability of a compound to donate an acidic proton to another reactive species in a specific medium. Although related experimental and theoretical approaches are based on reference acidity, the absolute approach may produce better results in terms of the medium in which one or more antioxidant components are found. This statement refers more to the theoretical approach, which better describes the intrinsic interactions rather than transferring them to the reference acidities. In contrast, experimental methods ensure that the (reference) acidity is accurate enough to assess the relationship between the acidity and scavenging activity [29,30,31], although some reports have concluded that such a general relationship is not observable [32]. The methods used for this purpose are usually based on liquid chromatography (either high-performance chromatography (HPLC) or standard chromatography (LC)) [33,34], in which suitable mobile phases are used to tune the interaction between the phase and the antioxidant compound. On the other hand, scavenging methods are usually formulated experimentally to serve as a reference. The standard antioxidant scavenging assay is based on the radical scavenging assay with 2,2-diphenyl-1-picrylhydrazyl (DPPH) [35,36]. Other assays widely used today are based on the reducing power of Cu(II) and Fe(III) ions (CUPRAC [37,38] or FRAP [39,40]), as well as assays that focus more on the total radical scavenging activity, such as oxygen (ORAC) [41,42], the hydroxyl radical absorbance capacity (HORAC) [43,44], and the Trolox equivalent antioxidant capacity (TEAC) [45,46]. For example, the ABTS (2,2’-azino-bis(3-ethylbenzothiazoline-6-sulfonic acid)) assay [47,48] was designed to be similar to the DPPH assay, with only minor differences in the topography of DPPH and ABTS, whereas ABTS may be considered more “sustainable” in terms of the reactivity of two chemical species.

**Scheme 1:**
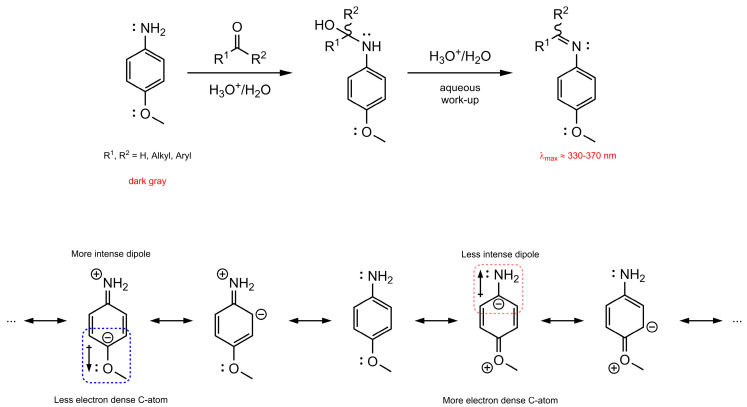
Imine formation with p-Anisidine as the reagent (**top**). The proton is present in the catalytic concentration and drives the reaction to the product. On the other hand, the resonance structures shown below exhibit a theoretical partial negative charge distribution with respect to the dipoles in the C−N and C−O bonds (as indicated in the schematic) [μ(=OMe^+^) = 2.523, μ(=NH_2_^+^) = 6.984, calculated at the mPW2PLYPD/def2-TZVP level of theory].

Theoretical insights into the structure and reactivity of antioxidants were attempted in the last decade when many random phase approximation (RPA)-based methods were published, including spin component (SC) and spin-opposite (SO) scaling algorithms for the specific treatment of spins in terms of spin correlation phenomena [49,50,51]. However, the thermodynamics and topography of antioxidants are still evaluated by calculations using generalized gradient approximation (GGA) type density functional methods (cf. DFT) rather than RPA methods. When combined with an appropriate basis set, GGA model chemistries show a fairly satisfactory level of accuracy, even in the calculation of antioxidant activity, polarizability (second derivative over energy) [52,53], and hyperpolarizability (third derivative over energy) [52]. The analytical assessments of intrinsic binding (the bond order, bond dissociation enthalpy (BDE), and forces on nuclei) also showed significant improvement in accuracy, although there is room for further corrections in the existing chemical models. The theoretical methods under development take into account some experimental backgrounds and justifications that are not yet adequately explained. One such example is the rationale for the p-Anisidine method for evaluating oxidative processes [54,55,56]. The electronic structure and topography of p-Anisidine do not provide sufficient data for a mechanism that could be considered experimentally relevant to this method. The most important point here is the difference in the electron density and resonance force of amino and methoxy substituents in the para position, which shows that the amino group at its nitrogen atom has a greater nucleophilic character that allows the formation of imines (Schiff bases) with the secondary products of lipid peroxidation (for theoretical treatment of this phenomenon, see [57]). This could be well described by observing the resonance models of p-Anisidine and measuring the dipole moments on certain zwitterionic species (Scheme 1). From now on, similar microchemical properties could be well evaluated using current chemical models, and precise discrepancies could generally be estimated and explained using quantum mechanical calculations.

Careful consideration of the brief theoretical aspect of the antioxidant theory mentioned above might lead one to conclude that, given a sufficiently accurate choice of theory, theoretical methods could provide sufficiently accurate data for some compounds and systems where experiments might lead to some experimental design difficulties, and that this might be the main reason for the use of theoretical methods in modern agricultural and food science. This was indeed the case; an extension of this approach occurred in the 2010s when the importance of quantum mechanical effects and the microchemical aspects of plants and food, in general, became clear [58,59]. Moreover, the development of theoretical methods allowed the better study of the mechanisms, thus promoting the incorporation of thermodynamic analyses in food science. Furthermore, the incorporation of theoretical methods into food science provided additional insight into newer mechanisms of antioxidant activity, such as radical adduct formation (RAF) and proton-coupled electron transfer (PCET). In addition, theoretical methods provided insights into the classical mechanisms of antioxidant activity, such as hydrogen atom transfer (HAT) [60,61,62] and sequential proton loss electron transfer (SPLET) [61,62].

Theoretical chemistry nowadays relies on ab initio methods (wavefunction theory, WFT) and DFT, where WFTs are much more expensive than DFTs, although WFTs are still ahead of DFTs on the accuracy scale, especially when it comes to post-Hartree-Fock methods (HFs) (e.g., Møller-Plesset or coupled cluster methods). On the other hand, the time requirements of DFT methods, which tend to reproduce MP2-level results, are much more favorable than those of WFT methods. This leaves an interesting gap in the discussion of the optimal balance between accuracy and time for classical calculations for small (up to ~30 atoms) and medium (approximately ~30–70 atoms) systems. It might be generally observed that the calculation of second derivatives (frequencies and Hessian evaluation) or third derivatives (hyperpolarizabilities) requires much more time to successfully complete the calculation and gradient evaluation than is the case for geometry optimizations (sometimes referred to as the minimum energy path (MEP) or nominal first derivatives). Various structures exhibit different stereochemical constraints; therefore, this general observation may vary with system topology. Nevertheless, since then, many different methods have been tested and proposed to achieve an optimal balance between accuracy and time within the DFT formalism, including variations based on electron interaction phenomena (i.e., electron exchange and correlation energies), which were uniquely described qualitatively by Perdew through the introduction of what he called Jacob’s ladder leveling [63]. The ladder consists primarily of the local spin density approximation (LSDA), the lowest level of the ladder, and proceeds to the generalized gradient approximation (GGA), then to the meta-GGA, and finally to the hyper-GGA methods, which include part of the exact Hartree-Fock exchange formalism. In recent updates of the ladder, there are random phase approximations (RPAs) [64] that may include a special case, double hybrid density functionals (DHDFs), which are constructed in a rather interesting way; it is a composite formalism empirically parameterized for the HF exact exchange part as well as the MP2-like (the so-called perturbative second-order theory, PT2) exact correlation (for more information see [65,66]). In summary, according to the current computational experience, the quality of the geometry parameters for the different theories (hereafter interchangeable with functionals) along the ladder is quite similar, with small improvements in the first derivative in terms of the coordinates as one moves up to RPA, which offers the possibility to work at lower theory levels (e.g., meta-GGAs or hyper-GGAs), while thermodynamic calculations and frequency estimates are more reserved for the higher levels of theory (e.g., hyper-meta-GGAs or RPAs). However, since the accuracy of the frequency calculation, in combination with the accuracy of the symmetry matrix during the evaluation, determines the thermodynamic accuracy, the scaling of frequencies may be important throughout the computational process, with the RPA and hyper-meta-GGA functions offering scaling factors closer to unity compared to the hyper-GGA and meta-GGA functions. This is also one of the reasons why general experience recommends performing optimizations at lower theory levels in conjunction with subsequent energy calculations at much higher theory levels.

## 2. Theoretical Background and Concept Description

An interesting question at this point might be: “Why is all this so important that it is emphasized?”. In the previous section, we briefly explained why such a “normal” calculation setup is prescribed for energy optimization calculations; therefore, a reverse approach to the calculation setup could also be proposed. In this approach, we relied on such a setup that could reduce the accuracy and time ratio to below optimum in such a way that the accuracy level is roughly maintained while the computational cost is significantly reduced (i.e., less time is required to compute the first and second derivatives). This led to the idea of more closely examining the current DFT functionalities of the second, third, and fourth rungs of Jacob’s ladder in order to swap them out to achieve the desired accuracy. Such a theoretical inversion was called inversion theory leveling (ITL). The key idea of ITL was to use functions of a more complex theoretical construction for the evaluation of the first derivatives (i.e., the minimum energy path (MEP) optimization process), i.e., to incorporate more diverse mathematical terms to describe electron exchange and correlation, with the subsequent use of such force constant estimation as an initial Hessian for the evaluation of the second derivatives at a lower level of theory. This key idea is generalized only in this way since the total computational effort is determined by the evaluation of the range of basis functions used to describe the behavior of individual electrons. Furthermore, the key idea should not be taken lightly at this point in the discussion because it is based on the principle of involving many-parameter participation. This is closely related to the general structure of a medium or even a small system, which may exhibit additional phenomena within its topography. Therefore, it is important to critically and thoroughly evaluate the topography prior to the ITL assessment. In general, interactions over long distances are considered less important or biased and neglected in the theoretical setup since interactions over short distances are considered stronger and more important for the evaluation of the critical force constant at the atomic level (i.e., the evaluation of ${\left(\frac{d^{2}E}{{\mathrm{dx}}^{2}},\frac{d^{2}E}{{\mathrm{dy}}^{2}},\frac{d^{2}E}{{\mathrm{dz}}^{2}}\right)}_{r,V}$, where V is the external potential and **r** is the interatomic radius). In contrast to this statement, several DHDFs have been pointed to long-range corrections associated with excited states and degeneracies that have been parameterized to treat spin components well [67,68]. The development of the above general idea in conjunction with the previously presented concepts in theoretical approaches primarily inspired the idea to develop the ITL approach, which was rationalized in two steps:

 *Step 1:*  Use more complex functionals with higher quality (i.e., triple or quadruple ζ-valence levels) for the MEP procedure;
 *Step 2:*  Apply moderately complex theories to obtain a good estimate of the frequencies and hence the thermodynamics. In the case of ITL, the refined differentiation of functionals had to be rewritten and improved. Moderately complex theories should include at least two complementary effect-parameterized terms in the mathematical structure of a specific function (i.e., the dispersion forces, BSSE correction for vicarious orbital radii, long-range treatment, etc.).

**Figure 1:**
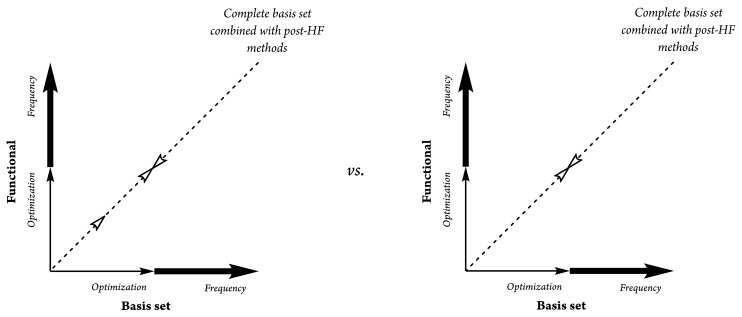
Graphical representation of the difference between the three-step corrected (**left**) and the standard (two-step corrected) approach of the ITL methodology (**right**). The hollowed arrows indicate the degree of theory extension or loss depending on the step (the same notation is used in Figure 2).

Following this logic, a multi-stage ITL could also be envisaged for implementation. This would involve an additional optimization step before step one, which would be evaluated at a lower level of theory, while the force constants of such an optimized structure would be retained for another optimization step, which would involve complex functionals with a lower quality basis set. In practice, this change in the basis set may be observed separately and generally leads to the additional refinement of the results in step two, as shown in Figure 1. On the other hand, in formulating the two steps required for ITL completion, one could see that some potentially effective examples for step one are functionals within the M06 and M05 families [69,70], as well as, albeit with some caution, mPW2PLYP, B2PLYP, and PBE-QIDH could be potentially good starting choices [71,72,73]. For step two, the best choice might be in the ωB97 family since there is perhaps the widest range of optimized effects for this family of functions (including ωB97, ωB97X, ωB97X-D, ωB97X-V, ωB97M-D, and ωB97M-V). On the other hand, empirical results on different systems have shown that functionals using PBE correlation functions could also provide good estimates of the frequencies, which emphasizes that a one-parameter hybrid of the PBE family (PBE0/PBEh) [74] could provide the best results on the second derivatives of different antioxidant systems.

**Figure 2:**
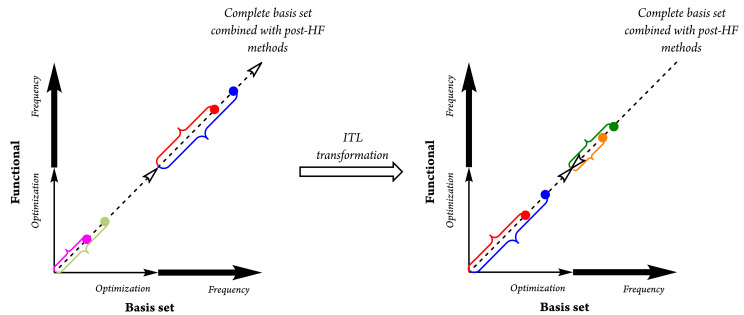
Graphical interpretation of the difference between the standard functional optimization step for frequency computation (**left**) and the ITL methodology approach (**right**). For the optimization process, the selected theory level B3LYP/6-311G(d,p) (the purple and dark yellow dots, respectively, corresponding to the functional/basis set notation) was used, with the subsequent use of M062X/cc-pVQZ (the red and blue dots, respectively) for the frequency calculation to describe the standard optimization path. In contrast, the ITL methodology was described by the inversion in theory used; the same M062X/cc-pVQZ was used for the optimization process, while ωB97X-V/pcseg-1 (the green and orange dots, respectively) was chosen as the level of theory for the frequency calculation in this type of approach. The theories mentioned in this heading are exemplary.

When used as a multi-step ITL, step one should include some GGA or meta-GGA functions, such as TPSS or M11L, followed by the second step with a hyper-meta-GGA theory level function (e.g., M052X). The third step should be in the same framework as the standard ITL. Figure 2 graphically summarizes the differences between ITL and the classical approach (also called the proportional approach).

Our attempts to implement ITL were aimed at developing increased accuracy for some functionals when used in this way. Namely, several DFT functionals of different hierarchies were chosen for testing based on their mathematical construction: B97 [75], a pure GGA functional with a third-generation dispersion correction with Becke-Johnson damping, an Austin-Peterson-Frisch hybrid functional [76] with an atomic sphere-based adjustment of the dispersion parameters, an M08HX hybrid meta-GGA functional [77], Grimme DHDF with benchmarking against non-covalent interactions, mPW2PLYP with first-generation dispersion corrections implemented, the Head-Gordon hybrid functional with an exact long-range exchange term and without an exact short-range exchange term (ωB97) [78], and in 1998, a later modification of the well-known one-parameter hybrid functional of Perdew, modified for particle-hole interactions in excited states, PBEh1PBE (we use a new acronym for this hybrid functional, i.e., PBEh0) [74]. In addition, three composite three-body functionals introduced by Grimme were included in this benchmark to account for the effects of the dynamical inversion of the basis set and function on the thermodynamics of the antioxidant species, i.e., r^2^SCAN-3c, B97-3c, and PBEh-3c. The first two are linked to the analogous pure meta-GGA and pure GGA functions, respectively, while the latter function is derived from an analogous PBE0 hybrid [79,80,81]. The starting antioxidant molecule was selected as the simplest phenol.

## 3. Results and Discussion

**Scheme 2:**
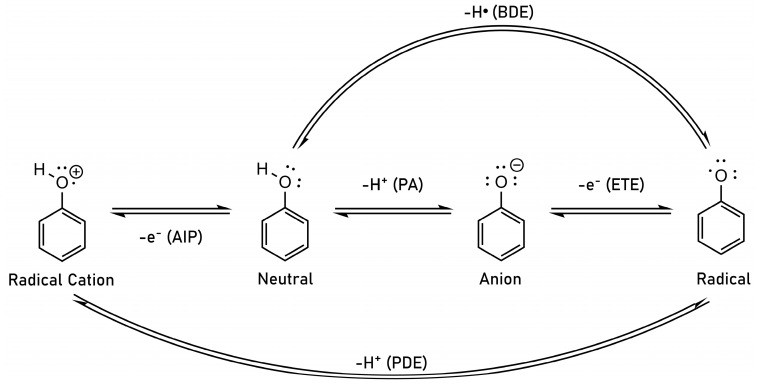
Graphical representation of the antioxidant scavenging processes associated with Equations (1)–(3) (a phenol is used as an example): BDE; bond dissociation enthalpy, PA; proton affinity, ETE; electron transfer enthalpy. Two additional processes are also presented here to fully explain the parameters for antioxidant scavenging activity: the proton dissociation enthalpy (PDE) and adiabatic ionization potential (AIP).

In order to properly and correctly set up the ITL methodology, the first step was to fully optimize the phenol (the fully relaxed structure) and calculate the frequencies with the correct functionals mentioned above, evaluating a lower quality basis set (i.e., double-ζ) for the polarization phenomenon with segmented exponents (pcseg-1) [82]. The only exceptions for this step were three-body composites, for which modified Weigend basis sets of double-ζ and triple-ζ quality, namely, def2-mSVP and def2-mTZVP/def2-mTZVPP, were created. The second step was to test the regular ITL approach by extracting the geometrically fully relaxed structure of the specific reactive phenol species and subjecting it to its analogous frequency calculations. The second step required the use of two different functionals to fully account for the ITL methodology as described above: LC -ωHPBE [83] and ωB97X-V(D3BJ) [84]). As seen, both are long-range corrected hybrid GGAs. The only difference is how the two treat the short-range interactions, for which we could then say that they both satisfy the general step two condition (see above). Next, we compared the bond dissociation enthalpies (BDEs), proton affinities (PAs), and electron transfer enthalpies (ETEs) chosen as the parameters for antioxidant activity, apart from the ionization potential (either adiabatic or vertical). Equations (1)–(3) briefly show how the BDEs, PAs, and ETEs were mathematically calculated, together with an additional schematic representation of the above processes in Scheme 2. After evaluating the three parameters, three levels of accuracy were introduced to better reveal the true nature of the ITL and these functionals; the introduction of a reference method at a higher level of theory, enthalpies compared to the G4(MP2) compound method [85], and experimental data for some of them to monitor the behavior of the ITL. Since all the scavenging effects of antioxidants are observed in the condensed phase, an implicit solvation model based on a conduction-like polarizable continuum model (CPCM) [86] was chosen for benzene.

(1) $$\mathrm{BDE}=\left(∆H_{\mathrm{radical}}^{298}+∆H_{H^{∙}}^{298}\right)\;-\;∆H_{\mathrm{neutral}}^{298}$$

(2) $$\mathrm{PA}=\left(∆H_{\mathrm{anion}}^{298}+∆H_{\mathrm{proton}}^{298}\right)\;-\;∆H_{\mathrm{neutral}}^{298}$$

(3) $$\mathrm{ETE}=\left(∆H_{\mathrm{radical}}^{298}+∆H_{\mathrm{electron}}^{298}\right)\;-\;∆H_{\mathrm{anion}}^{298}\;≅\;∆H_{\mathrm{radical}}^{298}-\;∆H_{\mathrm{anion}}^{298}$$

The reference method at a higher theory level was chosen based on the results of Galano and co-workers [87], where it was found that the theory level M052X/6-311+G(d,p) could produce the best results so far. However, even if accuracy is important, the total time (computational cost) is an important parameter in the choice of the function. This was the main argument for our approach to reduce the quality of the basis set for a ζ-, i.e., the polarization level, in order to save time for the same computation. Finally, both the property parameters (BDE, PA, and ETE) and the wall times were monitored, compared, and subjected to basic statistical treatment. Their relative deviation was measured, referred to here as the relative absolute deviation (RAD), and calculated as follows:

(4) $$\mathrm{RAD}=\frac{\left|x_{0}{\;-\;x}_{\mathrm{ref}}\right|}{x_{\mathrm{ref}}}100\Leftrightarrow \frac{\left|∆H_{\mathrm{parameter}}^{\mathrm{functional}}-∆H_{\mathrm{parameter}}^{\mathrm{experimental}}\right|}{∆H_{\mathrm{parameter}}^{\mathrm{experimental}}}100$$

The results in Table 1 strongly suggest that the functionals generally described at a higher level of Jacob’s ladder provide significantly better results for all three parameters chosen, although it is questionable whether the total wall time parameter could be considered acceptable or fits within the acceptable range of optimal parameters. A two-step comparison (i.e., the RAD for the tested functionals based on the experimental values) was simultaneously compared with the analogous benchmarks against the functionals with high general accuracy (the reference functionals mentioned above) (Table 2) would clarify the perhaps surprising claim that some hybrid DFT functionals could perform above average in such a test against specific references. A similar agreement with the experimental results was observed for the proposed deviations (RADs) for the same hybrid DFT functionals, in particular for the meta-GGA hybrid DFT M08HX and the DHDF type mPW2PLYPD. Performing standard first and second gradient evaluations for these two tested theory levels leads to the clear conclusion that their BDEs range from 84 to 87.7 kcal/mol upon implicit solvation. The results strongly suggest a positive trend between the BDEs in the gas phase and implicit solvation, in conjunction with the experimental results for these types of parameters. A similar trend was observed for the proton affinities, suggesting that these two DFT functionalities could be used to evaluate antioxidant activity. However, the electron transfer enthalpies differed slightly from the experimental values, which we believe leaves room for further discussion on the accuracy of these two functionalities. Nevertheless, the ETE itself might be a somewhat speculative parameter, mainly because of the process of electron solvation, as well as its accuracy and, ultimately, the need to include this parameter in Equation (3).

Although the RAD, like quantity, is quite sensitive to small changes in the values in the tested functionals, we tend to believe that the increased RADs for the ETEs for these two functionals do not significantly affect the accuracy of these functionals. In addition, a surprising phenomenon occurred when they were subjected to the ITL approach. As predicted in the previous sections, ITL may be sensitive to changes in the accuracy of the species for which the thermodynamic parameters were calculated or to the error introduced by the theory used in these calculations. Therefore, a positive trend is observed for all functionals except M08HX and mPW2PLYPD. Of particular note are the composite three-body methods, whose performance was much worse than the others. In accordance with the ITL postulate, the change in the behavior of these three functionals was significant when applied according to the ITL principle, reducing their RAD from 12% to 5–7%.

According to the ITL background theory proposed above, we assumed that three-body composite methods together with M08HX and mPW2PLYPD should be considered for ITL testing purposes only for the optimization process. In the second step, the choice of functionals was more difficult, although the selection was reduced to hybrid GGA functionals since they are halfway on Jacob’s ladder, considering the theoretical complexity. Finally, as mentioned above, two long-range corrected functionals were selected. Table 3 shows the results of the ITL method using the LC-ωHPBE functional. Interestingly, all parameters and, consequently, the RADs showed a tremendous intensity of the leveling process in the overall results so that all functionals achieved practically the same level of accuracy regardless of their theoretical structure or complexity. The computational times for such calculations were only slightly increased compared to the original methods. The leveling that occurred when using the LC-ωHPBE functional could be due to an insufficient empirical ratio of the coefficients in the short and long range exchange regions in screening.

Table 4 shows that ωB97X-V(D3BJ) produced significantly better results under ITL treatment than LC -ωHPBE. An original conjecture that could be defined as a mere solution to the potential dilemma of why the ωB97 class functional performs better than LC—one could either consider a sophisticated mathematical apparatus for ωB97 likely, considering that it was constructed based on the pure B97-GGA functional, while the percentage of exact exchange in the short-range HF was parameterized differently, with a slightly lower percentage in ωB97X-V(D3BJ), which is ~16%. The leveling effect also disappeared when ωB97X-V(D3BJ) was used with some improvement in three-parameter accuracy, especially for the composite three-body functionals. In addition, the performance of this functional in M08HX and mPW2PLYP deteriorated slightly compared with the results of the original SCF calculations, but these results did not exceed the expected deviation of more than 2–3% in the RAD, indicating that ITL is a very suitable theoretical approach for calculations related to antioxidant activity.

Therefore, the reference method M052X/6-311+G(d,p) proved to be a very accurate theoretical level for the calculation of antioxidant parameters, although its total wall time was much larger than that of the other methods used or the ITL method. As with the ITL method and all other methods tested, the use of the pcseg-1 basis set also showed a significant decrease in the estimated total wall time. This suggests the use of ITL in further analyses, as well as the use of the meta-GGA hybrid functionals and DHDFs mentioned above for the same type of calculations. Two specific comments about accuracy should be specified at this point:

1. M08HX performed almost as well as the reference method and provided the antioxidant parameters mentioned here. A similar line could be drawn for the total wall times, which are almost twice as fast as the reference method.
2. Apart from the leveling effect, the ITL method improved results in usually low-cost methods, such as the three-component composite methods presented in this manuscript.

Summarizing the above performances, the results reported in the BDEs of the selected theoretical methods showed a moderate to poor agreement for low-cost composite methods with RADs of approximately 6–11 kcal/mol, while the hyper-GGA methods generally performed well, with RADs of ~ 3–4 kcal/mol. The DHDF and hyper-meta-GGA methods showed superior accuracy in their BDEs, which exceeded chemical accuracy, with a RAD of ≤ 1 kcal/mol. On the other hand, the PA and ETE showed very good agreement, even for low-cost composite methods, with a RAD of 1.5–2.5 kcal/mol for the PA and 2–9 kcal/mol for the ETE, in general. This should indicate an important property of the presented low-cost methods, i.e., their general efficiency and rapidity. On the other hand, as mentioned above, ITL outperformed all the selected methods, including DHDF and hyper-meta-GGA. This leveling phenomenon should be reasonable considering the anchoring method for the frequency calculation (ωB97X-V(D3BJ) and LC -ωHPBE). The choice of the reference anchoring method should also be carefully considered when performing ITL since the two chosen methods showed discrepancies in both their thermodynamics and wall times.

Moreover, regarding the methodology presented herein, we also propose to use a special ITL notation for ITL in future studies that include two backslashes to emphasize the inverse nature of the theory levels used, as mentioned in Table 3 and Table 4.

## 4. Conclusions and Outlook

Inversion theory leveling, as a recently modified approach, has opened an interesting perspective for thermodynamic calculations based on the results presented, linking theoretical chemistry to antioxidants and antioxidant activity by pointing either to the parameters of antioxidant activity or to the structure of antioxidants and their intrinsic reactivity on which current computational methods in chemistry are based. The overall performance in terms of thermodynamics and wall times relevant to antioxidants in a computational sense highlights the importance of the ITL method for future studies and also renders it a very flexible method for broader chemical studies. In the field of food science, ITL could be a promising theoretical tool to investigate the quality of theoretical calculations, leading to the development of new perspectives in theoretical chemistry as well as in food science and antioxidant theory.

## 5. Computational Details

Optimizations and frequencies were performed using Gaussian16 [92] for all functionals except for the composite three-body functionals (PBEh-3c, B97-3c, and r2SCAN-3c), which were performed using Orca 5.0.1 [93]. Gaussian16 was also used for the LC -ωHPBE ITL frequency calculations, while the frequency calculations at ωB97X-V(D3BJ) were performed using Orca 4.2.1 [94]. The calculations were performed in the gas phase and the implicit solvation model using the CPCM approach for benzene as the chosen solvent. In addition, the SCF energy threshold of 10^−12^ Eh was set for all calculations, and the highest integral grid was used, i.e., INT = SUPERFINE. A segmented polarization-modeled basis set of double-ζ quality (pcseg-1) was chosen as the common basis set.

## Figures and Tables

**Table 1:** The BDEs, PAs, and ETEs calculated using the original method approach, evaluating the first and second derivatives for each level of theory (in kcal/mol).

Original Method	BDE		PA		ETE	
	GP	ISM/CPCM	GP	ISM/CPCM	GP	ISM/CPCM
B97(D3BJ)	81.033	80.996	348.769	261.125	47.928	76.262
ωB97	81.657	81.715	348.983	260.808	47.664	76.624
M08HX	87.462	87.571	346.680	258.620	56.408	85.302
APFD	82.045	82.168	348.185	260.147	49.376	78.263
PBEh0	81.341	81.467	347.802	259.740	48.564	77.478
mPW2PLYPD	84.195	84.107	349.035	261.079	48.048	76.642
B97-3c	77.206	81.492	346.759	258.697	49.298	78.019
r^2^SCAN-3c	81.961	80.331	345.474	256.885	49.274	78.670
PBEh-3c	80.275	79.011	349.590	261.013	43.207	73.223
M052X/6-311+G(d,p)	87.695	87.537	347.570	259.038	54.130	83.228
G4(MP2)	85.696	85.209	346.795	258.797	53.967	82.951
Experimental	88.340 ^a^	87.200 ^b^	347.99 ^c^	-	52.10 ^d^	-

^a^ [88] ^b^ [89] ^c^ [90] ^d^ [91]

**Table 2:** Relative absolute deviations (RADs) for all original methods (in %) and their total wall times (in s).

Original Method	RAD						Wall Time		
	BDE (GP)	BDE (ISM)	PA (GP)	PA (ISM)	ETE (GP)	ETE (ISM)	GP	ISM/ CPCM	Total
B97(D3BJ)	8.27	7.12	0.22	-	8.01	-	1989.00	716.00	2705.00
ωB97	7.57	6.29	0.29	-	8.51	-	1123.00	1059.00	2182.00
M08HX	0.99	0.42	0.38	-	8.27	-	1233.00	1457.00	2690.00
APFD	7.13	5.77	0.06	-	5.23	-	700.00	914.00	1614.00
PBEh0	7.92	6.57	0.05	-	6.79	-	940.00	838.00	1778.00
mPW2PLYPD	4.69	3.55	0.30	-	7.78	-	1970.00	2084.00	4054.00
B97-3c	12.60	6.55	0.35	-	5.38	-	1571.48	1882.67	3454.15
r^2^SCAN-3c	7.22	7.88	0.72	-	5.42	-	1936.52	2113.98	4050.50
PBEh-3c	9.13	9.39	0.46	-	17.07	-	971.52	900.32	1871.84
M052X/6-311+G(d,p)	0.73	0.39	0.12	-	3.90	-	3816.00	5150.00	8966.00
G4(MP2)	2.99	2.28	0.34	-	3.58	-	9915.00	31827.00	41742.00

**Table 3:** The BDEs, PAs, and ETEs (kcal/mol), their respective estimated RADs (%), and the total wall times (s) performed using the *original method/pcseg-1\\LC -ωHPBE/pcseg-1*.

Original Method\\LC-ωHPBE	BDE		PA		ETE		RAD						Wall Time		
	GP	ISM	GP	ISM	GP	ISM	BDE (GP)	BDE (ISM)	PA (GP)	PA (ISM)	ETE (GP)	ETE (ISM)	GP	ISM	Total
B97(D3BJ)	81.501	81.663	350.713	262.402	48.697	77.896	7.74	6.35	0.78	-	6.53	-	1202.00	1505.00	2707.00
ωB97	81.560	81.695	350.672	262.363	48.797	77.966	7.68	6.31	0.77	-	6.34	-	1420.00	1178.00	2598.00
M08HX	81.548	81.681	350.681	262.374	48.776	77.942	7.69	6.33	0.77	-	6.38	-	1100.00	1092.00	2192.00
APFD	81.585	81.723	350.653	262.350	48.841	78.008	7.65	6.28	0.77	-	6.26	-	904.00	1011.00	1915.00
PBEh0	81.624	81.754	350.656	262.347	48.877	78.041	7.60	6.25	0.77	-	6.19	-	805.00	1060.00	1865.00
mPW2PLYPD	81.582	81.718	350.681	262.372	48.810	77.981	7.65	6.29	0.77	-	6.31	-	2400.00	2537.00	4937.00
B97-3c *	78.920	78.920	347.833	259.434	48.996	78.122	10.66	9.49	0.05	-	5.96	-	1213.89	1323.30	2537.19
r^2^SCAN-3c *	78.796	78.796	347.882	259.455	48.823	77.976	10.80	9.64	0.03	-	6.29	-	1420.54	1597.42	3017.96
PBEh-3c *	78.792	78.760	347.834	259.388	48.867	78.006	10.81	9.68	0.04	-	6.21	-	777.95	612.29	1390.24

* These methods are modeled to perform with their own modified def2-type basis set.

**Table 4:** The BDEs, PAs, and ETEs (kcal/mol), their respective estimated RADs (%), and the total wall times (s) performed using the *original method/pcseg-1\\ωB97X-V(D3BJ)/pcseg-1*.

Original Method\\ω-B97X-V(D3BJ)	BDE		PA		ETE		RAD						Wall Time		
	GP	ISM	GP	ISM	GP	I SM	BDE(GP)	BDE (ISM)	PA (GP)	PA (ISM)	ETE (GP)	ETE (ISM)	GP	ISM	Total
M08HX	85.301	85.502	352.981	263.967	48.885	79.635	3.44	1.95	1.43	-	6.17	-	1886.86	2387.85	4274.71
PBEh0	85.252	85.448	352.948	263.907	48.869	79.641	3.50	2.01	1.42	-	6.20	-	1701.47	1810.67	3512.14
mPW2PLYPD	85.296	85.500	352.976	263.924	48.885	79.675	3.45	1.95	1.43	-	6.17	-	2706.85	2580.22	5287.07
B97-3c *	82.010	82.103	350.201	261.038	48.375	79.164	7.17	5.85	0.64	-	7.15	-	2005.54	2279.77	4285.31
r^2^SCAN-3c *	82.573	82.671	350.270	261.106	48.867	79.665	6.53	5.19	0.66	-	6.21	-	2272.68	2492.85	4765.53
PBEh-3c *	81.851	81.940	350.194	261.078	48.223	78.962	7.35	6.03	0.63	-	7.44	-	1991.85	1574.31	3566.16

* These methods are modeled to perform with their own modified def2-type basis set.

## Data Availability

Not applicable.
